# Biological response to Przewalski’s horse reintroduction in native desert grasslands: a case study on the spatial analysis of ticks

**DOI:** 10.1186/s12862-024-02252-z

**Published:** 2024-05-11

**Authors:** Yu Zhang, Jiawei Liu, Ke Zhang, Anqi Wang, Duishan Sailikebieke, Zexin Zhang, Tegen Ao, Liping Yan, Dong Zhang, Kai Li, Heqing Huang

**Affiliations:** 1 School of Ecology and Nature Conservation, Beijing Forestry University, Beijing, China; 2 Northwest Institute of Plateau Biology, Chinese Academy of Science, Xining, China; 3 Xinjiang Fuyun County Kizillike Township Agricultural Development Center, Altay, China; 4 Tongliao Forestry Pest Control Station, Tongliao, China; 5 Tongliao Control and Quarantine Station of Forest Pest, Tongliao, China; 6 Chongqing Academy of Environmental Science, Chongqing, China

**Keywords:** Reintroduction ecology, Arid desert area, *Hyalomma asiaticum*, Przewalski's horses, Spatial distribution, Host and parasite interaction

## Abstract

**Background:**

Reintroduction represents an effective strategy for the conservation of endangered wildlife, yet it might inadvertently impact the native ecosystems. This investigation assesses the impact of reintroducing endangered Przewalski's horses into the desert grassland ecosystem of the Kalamaili Nature Reserve (KNR), particularly its effect on the spatial distribution of ticks. In a 25 km^2^ core area of Przewalski's horse distribution, we set up 441 tick sampling sites across diverse habitats, including water sources, donkey trails, and grasslands, recording horse feces and characteristics to analyze the occurrence rate of ticks. Additionally, we gathered the data of 669 fresh feces of horses. To evaluate the spatial dynamics between these feces and ticks, we used methods such as Fixed Kernel Estimation (FKE), Moran’s *I* spatial autocorrelation index, and Generalized Linear Models (GLM).

**Results:**

The dominant species of ticks collected in the core area were adult *Hyalomma asiaticum* (91.36%). Their occurrence rate was higher near donkey trails (65.99%) and water sources (55.81%), particularly in areas with the fresh feces of Przewalski's horses. The ticks’ three risk areas, as defined by FKE, showed significant overlap and positive correlation with the distribution of Przewalski's horses, with respective overlap rates being 90.25% in high risk, 33.79% in medium risk, and 23.09% in low risk areas. Moran's *I* analysis revealed a clustering trend of the fresh feces of Przewalski's horses in these areas. The GLM confirmed a positive correlation between the distribution of *H. asiaticum* and the presence of horse fresh feces, alongside a negative correlation with the proximity to water sources and donkey trails.

**Conclusions:**

This study reveals the strong spatial correlation between Przewalski's horses and *H. asiaticum* in desert grasslands, underlining the need to consider interspecific interactions in wildlife reintroductions. The findings are crucial for shaping effective strategies of wildlife conservation and maintaining ecological balance.

**Supplementary Information:**

The online version contains supplementary material available at 10.1186/s12862-024-02252-z.

## Background

Desert grassland ecosystems are a critical component of terrestrial ecosystems, harboring unique communities of flora and fauna adapted to extreme arid conditions [1, 2]. Nonetheless, the ungulates in these ecosystems, serving as pivotal species for maintaining ecological balance, are confronting a series of challenges including habitat loss and climate change [3, 4]. In this context, endangered species reintroduction stands as one of the effective measures for the restoration and preservation of desert grassland ecosystems, as well as for augmenting their biodiversity [2, 5, 6]. The successful reintroduction of the Przewalski’s horse into the Kalamaili Nature Reserve (KNR) in Xinjiang, China, serves as a quintessential example of this practice [7, 8]. The KNR is located at the southern edge of the Junggar Basin in northwestern China, characterized by an arid climate and infrequent rainfall, leading to the formation of a unique Central Asian continental desert grassland biota [8, 9]. Since 2001, the population of Przewalski's horses in the region has experienced substantial growth, escalating from 27 to 230 by the year 2019 [8–10]. This resurgence of this population in the KNR provides us with a unique opportunity to explore the responses of related species following the reintroduction of large ungulates into their native ecosystems.

However, as an introduced species, the reintroduction of Przewalski's horses could potentially give rise to some unforeseen ecological consequences, especially regarding the impact on parasite dynamics [10–12]. In response, a specialized team undertakes annual monitoring of parasitic diseases within the Przewalski’s horses [10, 12–14]. It was discovered that the Przewalski's horses in the KNR are heavily infected with the obligate parasite, *Gasterophilus* spp. [10, 14], with infection intensity more than double that found in the sympatric Mongolian wild ass (*E. hemionus*) [13]. The research team initially aimed to study the transmission patterns of the myiasis disease by tracking and examining the feces of Przewalski's horses, as the larvae of these *Gasterophilus* spp. tend to burrow into the soil within 5 min after the horse's defecation [10–14]. However, in recent investigations, we inadvertently found that ticks frequently appeared near the feces of the Przewalski's horses, which sparked our interest in studying the spatial distribution relationship between Przewalski's horses’ feces and ticks. Additionally, during the peak season of tick activity in spring and summer [15, 16], we incidentally discovered the *Hyalomma asiaticum* (Acari: Ixodidae) parasitizing on the abdomen of deceased Przewalski's horses (Fig. S1), and also fortuitously encountered naturally detached and engorged adult *H. asiaticum* near the Przewalski's horse dung piles (Fig. S2). These observations not only corroborate the hypothesis that Przewalski's horses could serve as potential new hosts for *H. asiaticum* but also provide important clues into the interactions between hosts and parasites in the KNR.

*H. asiaticum*, a tick prevalent in arid desert regions, poses a direct threat to host animals through biting, leading to conditions such as inflammation and anemia [17–20]. It also plays a crucial role in the transmission of various diseases, including Crimean-Congo Hemorrhagic Fever (also known as Xinjiang Hemorrhagic Fever locally) and Rickettsial diseases, among others [21, 22]. Being a three-host parasitic tick, *H. asiaticum* progresses through a life cycle consisting of four stages: egg, larva, nymph, and adult [15]. Parasitism occurs in the stages after the egg, with immature *H. asiaticum* ticks predominantly parasitizing small rodents, and adults typically attaching to large ungulates such as horses, cattle, and sheep [23–27]. Ticks generally employ two strategies to seek hosts: the ambush strategy and the hunter strategy. In the ambush strategy, ticks usually position themselves on the top of plants or rocks, waiting for hosts to pass by. Conversely, in the hunter strategy, ticks move through the environment, actively seeking out hosts by detecting carbon dioxide released by the hosts and following trails of the hosts’ excreta [28, 29]. *H. asiaticum* are typical hunter type ticks, as they conceal themselves in the surrounding environment and actively wait to attack passing hosts [15, 28, 30]. Notably, the adult *H. asiaticum* in the unfed state, while actively seeking host animals, not only demonstrate a strong preference towards large ungulate hosts but also amplify the risk of disease transmission during this process [15, 18]. Hence, understanding and monitoring the distribution of ticks at this specific life stage is paramount for the efficacious prevention and management of the pervasive transmission of tick-borne diseases in the KNR.

The water sources in arid and desert regions, along with their adjacent areas, are vital habitats for wildlife [31, 32]. In the KNR, the Przewalski's horses exhibit unique seasonal behaviors. In the spring and summer, they frequently travel along designated paths, known as 'donkey trails' [11], to reach water sources. They tend to congregate around these water sources and the adjacent grasslands, showing a pronounced propensity to cluster more notably than other ungulate species such as the Mongolian wild ass (*E. hemionus*) and the Goose-throated gazelle (*Gazella subgutturosa*) [7, 11, 33]. This behavioral pattern does not only highlight the attraction of water sources for Przewalski’s horses but also presents potential opportunities for the local parasite populations to spread [11, 12]. Although there is a notable correlation between the spatial distribution of wildlife and parasites [11, 12, 29, 34, 35], studies specifically examined the relationship between the distribution of Przewalski’s horses and parasites are comparatively limited [11, 12]. A previous study revealed that areas within a 300 m radius surrounding water sources, nortably high density of Przewalski's horse feces were observed [11]. Furthermore, there is a positive correlation between the density of Przewalski's horse feces and the spatial distribution of the *Gasterophilus* spp. [11, 12]. This further confirms the significance of water sources and their surrounding areas as principal locales for the interaction between Przewalski's horses and potential parasites. Moreover, a prior report indicated that before the reintroduction of Przewalski's horses into the KNR, large-scale tick infestations were not observed [16]. However, this historical absence of infestations does not rule out the potential for these horses to experience health challenges when reintroduced, as they may lack natural immunity or the ability to adapt to local parasites [11–13]. For instance, a decade after elk (*Cervus canadensis*) were reintroduced to southeastern Kentucky, USA, the distribution of local ticks became more widespread [36]. Similarly, in Japan, as populations of sika deer (*C. nippon*) and wild boar (*Sus scrofa*) increased, the distribution of the *Haemaphysalis* ticks changed, potentially increasing the transmission of the pathogen causing tick-borne diseases [37]. These findings highlight the importance of monitoring changes in local ticks distribution following reintroduction of wildlife [38, 39]. Therefore, this study focused on the Przewalski's horses and the ticks, conducted systematic surveys of typical habitats for horses in the KNR, including water sources, grasslands, and donkey trails. It incorporates the activity patterns of Przewalski's horses to thoroughly examine the spatial distribution relationship between host and parasite. The study aims to analyze the risk areas of ticks in the core distribution areas of Przewalski's horses. This study will provide a comprehensive assessment of whether reintroduced animals have expanded the distribution range of parasites, thereby offering a new perspective on understanding the synergistic adaptation of reintroduced species with related species in their new environment.

## Methods

### Research area

**Fig. 1:**
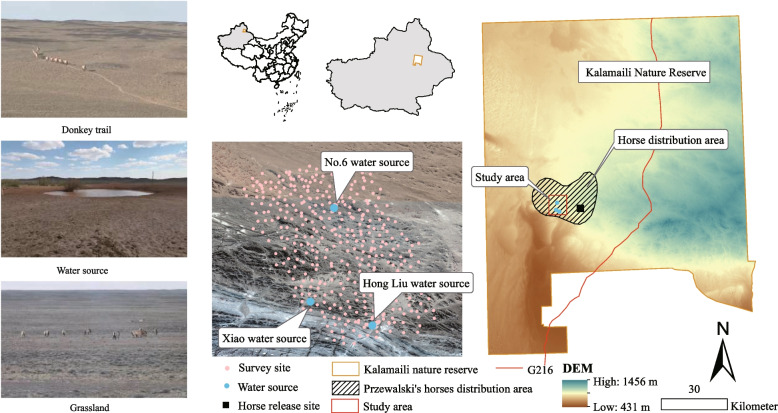
Tick sampling sites in Kalamaili Nature Reserve (KNR). Note: The left section delineates the three types of study habitats, namely, donkey trails, water sources, and grasslands; the center displays the distribution map of the sampling sites of ticks; the right section represents the study area

The KNR (88°30’ ~ 90°03’E, 40°36’ ~ 46°00’N) is located in the Junggar Basin of Xinjiang, China (Fig. 1), and is a typical arid and semi-arid desert grassland. The reserve has scarce water resources, with an annual precipitation of only about 159 mm [10, 40]. The composition of plant communities is relatively simple, featuring an average coverage of 20 to 30%, primarily consisting of xerophytic shrubs and herbs, such as *Ceratoides* spp., *Tamarix* spp., *Haloxylon* spp., *Anabasis* spp., and *Reaumuria* spp. [40]. Among them, the Hong Liu, Xiao, No.6 are key water sources within the region [11, 12]. Przewalski’s horses primarily depend on these water sources for their distribution, whereas other wildlife, including the *E. hemionus* and the *G. subgutturosa*, have a broad distribution throughout the entire reserve [7, 12, 31, 40]. Based on this context, this study was conducted during the season when is the peak of *H. asiaticum* activity from April to June 2021 [20, 41], focusing primarily on the spatial utilization patterns of Przewalski’s horses and their impact on the distribution of ticks. The research was centered around three water sources, designating a 25 km^2^ area as the study area, which also constitutes the core habitat of Przewalski’s horses (Fig. 1).

### Research method

#### Ticks sampling sites’ survey

Tick sampling sites were set up across three types of typical habitats: water sources, donkey trails, and grasslands, and the specific sampling method is shown in Fig. S3. In each habitat, based on the frequency of Przewalski’s horses’ activities, three types of dung piles were randomly selected for tick collection: stallion feces, non-stallion feces and no feces (Fig. S4). Stallion feces are generally higher than the feces of non-stallions because stallions repeatedly defecate in the same location, forming larger mounds. Conversely, non-stallion defecation behavior tends to be more random, usually occurring just once at various locations, resulting in smaller dung piles.

Arid desert regions are mainly covered with small shrubs. In such environments, ticks typically adopt an active waiting and attacking strategy for survival [28, 30].

Therefore, to ensure consistency in the sampling process, we limited time at each tick sampling site to 5 min, employing the 'waiting for ticks' method as referenced by Yu et al. [16]. The specific procedure involved shaking the ground with sticks in areas with *Tamarix* spp. and *Haloxylon* spp. to attract and collect non-engorged ticks [16, 28, 30]. Concurrently, each sampling site was centered within a defined area of 2 m × 2 m, thereby maintaining an effective sampling area of 4 m^2^. Using the “Create Buffer” and “Create Fishnet Tool” in ArcGIS 10.3, buffer zones were delineated, extending 100 m from the three water sources perimeters and 10 m along the donkey trails. Additionally, the grassland area was segmented into a grid pattern, each cell measuring 500 m × 500 m. The collected data were stored in KML format and subsequently imported into a GPS device (eTrex309x, Garmin Ltd., Olathe) to precisely locate the sampling sites for efficient field collection. Furthermore, at the sampling sites where dung piles were present, we conducted detailed measurements of each dung pile’s characteristics. The dimensions of the dung piles (length × width × height in cm) were recorded using a tape measure. The moisture content of these dung piles was assessed using a soil moisture meter (PR-ECTH-SC-37DC, Pruisen brand Ltd., China) with an accuracy of ± 2% RH. Based on these readings, moisture levels were classified into two categories: low moisture (0 ~ 15% RH) and high moisture (15 ~ 30%RH). This classification approach is based on previous experience and is consistent with the fecal moisture classification standards found in the literature [42, 43].

The classification and identification of the ticks, along with the tallying of their numbers and other pertinent details, were carried out in the laboratory. Using a SZ51 stereomicroscope equipped with LED lighting / SZ2-ILST (Olympus corporation, Tokyo, Japan), we scrutinized the distinguishing features of the ticks, which encompassed the dorsal surface, ventral surface, eye, basis capituli, scutum, porose area, spiracle plate, marginal groove, and genital groove [44–46].

#### Przewalski’s horses’ spatial distribution survey

The distribution and density of wildlife feces are reliable indicators of spatial utilization in designated areas [40, 47, 48]. For data collection, we tracked Przewalski’s horses, and recorded the GPS coordinates of fresh feces after the horses had departed from the area. Our survey encompassed 8 herds, comprising a total of 60 reintroduced horses [12]. The study area included frequently visited water sources and grasslands in the KNR, enveloping the same 20km^2^ area also surveyed for ticks [12].

### Data analysis

#### The occurrence rate of H. asiaticum under different conditions

1 $$\begin{array}{c}{P}_{ij}=\frac{{N}_{ij}}{{M}_{ij}}\times 100\%\end{array}$$

Formula (1), ${P}_{ij}$ represents the occurrence rate of ticks under different conditions, ${N}_{ij}$ is the number of sampling sites with ticks under different conditions, ${M}_{ij}$ is the total number of sampling sites under different conditions. $i$ is water source, donkey trail, grassland, and $j$ is stallion-feces, non-stallion-feces and no-feces.

#### Analysis of H. asiaticum occurrence rate and Przewalski’s Horse Dung Parameters

Given the observed higher occurrence rates of *H. asiaticum* near the dung piles of Przewalski’s horses, this study investigates the influence of the dung piles’ physical characteristics (size and moisture) on the occurrence rate of the *H. asiaticum*. Dung pile size parameters include three key dimensions: pile height (cm), bottom area (cm^2^), and volume (cm^3^). Concurrently, the moisture content of the dung piles is categorized into two levels: low moisture (0 ~ 15% RH) and high moisture (15 ~ 30%RH). For maintaining consistency and ensuring comparability among variables, the variables were standardized using the Z-score function from the R package scale. The relationship between the number of the *H. asiaticum* and dung pile size was analyzed through Pearson correlation analysis. A correlation coefficient (r) approaching 1 indicates a stronger correlation. T-tests were used to compare the size differences of dung piles with and without the presence of the *H. asiaticum*, using *p* < 0.05*, *p* < 0.01**, and* p* < 0.001*** as thresholds to ascertain the levels of statistical significance. By calculating the occurrence rates of the *H. asiaticum* at different moisture levels, this study visually presents the results using bar graphs. The plotting was conducted utilizing the ‘ggplot2’ package in R.

#### Risk area analysis of the H. asiaticum

Based on the analysis results from the occurrence rates of the *H. asiaticum*, this study selected the GPS locations where the *H. asiaticum* was found. Additionally, by referencing to the GPS locations of Przewalski’s horses fresh dung piles reported in Zhang et al. [12], a comprehensive assessment of the spatial distribution relationship between the *H. asiaticum* and the horse fresh feces. These data underwent preprocessing using the R packages ‘sp’, ‘sf’, and ‘tidyverse’, which included data cleaning and standardization to ensure accuracy and consistency. The R package ‘adehabitatHR’ was utilized to calculate the 95% Minimum Convex Polygon (MCP) for the fresh feces of Przewalski’s horses, establishing their distribution within the 25 km^2^ study area (Formula 2). The definition of risk areas is based on Fixed Kernel Estimation (FKE) to categorize the distribution of ticks into high, medium, and low risk levels. Specifically, 50% FKE is used to define high risk areas, 75% FKE for medium risk areas, and 95% FKE for low risk areas (Formula 3). This classification reflects the occurrence rate of *H. asiaticum* within the study area. The Intersect module in the Arctoolbox of ArcGIS 10.3 software was employed to evaluate the spatial overlap between the distribution of Przewalski’s horses and the three risk areas of the *H. asiaticum*.

MCP calculation formula:

2 $$S=\frac{{x}_{1}\left({y}_{n}-{y}_{2}\right)+{\sum }_{i=2}^{n-1} {x}_{i}\left({y}_{i}-1-{y}_{i}+1\right)+{x}_{n}\left({y}_{n}-1-{y}_{i}\right)}{2}$$

Formula (2), S is the area, and ${x}_{i}$ and ${y}_{i}$ are the latitude and longitude.

FKE calculation formula:

3 $$F(x)=\frac{1}{2\pi {nh}^{2}}{\sum }_{i=1}^{n}exp\left[\frac{{dist}_{i}^{2}}{{2h}^{2}}\right]$$

Formula (3), n represents the number of ticks, h is the bandwidth, and ${dist}_{i}$ represents the distance between the point i and the geographical coordinates.

#### Spatial autocorrelation analysis

Drawing from the findings of the risk area analysis of the *H. asiaticum*, this study conducted a bivariate spatial autocorrelation analysis of the spatial distribution of horse fresh feces within the three risk areas. We employed the nearest neighbor method to create spatial weight matrices and used these matrices to calculate the Cross Moran’s *I* index for each area [49, 50]. This index aims to evaluate the degree of clustering between the horse fresh feces and the risk distribution areas of the *H. asiaticum*. Specifically, the Moran’s *I* value approaching 1 indicates a high level of clustering of horse fresh feces in the area, while values close to 0 suggest a weaker spatial association with horse fresh feces.

#### Kernel density estimation and multiscale correlation analysis

The spatial density of the Przewalski’s horse fresh feces and the *H. asiaticum* was calculated using Kernel Density Estimation (KDE) in the Spatial Analyst module of ArcGIS 10.3 (Formula 4). In this study, a uniform pixel value of 20 and a search radius of 500 m [12], were configured to precisely evaluate the correlation between the Przewalski’s horse fresh feces and the *H. asiaticum* across multiple scales. Five scales, 100 m, 250 m, 500 m, 750 m, and 1000 m (Fig. S5), were selected for conducting Pearson correlation analysis. We used ArcGIS 10.3 to construct grids corresponding to the five scales and to generate a central representative point for each grid. Based on the KDE layers of the *H. asiaticum* and Przewalski’s horse fresh feces, we extracted the raster attribute values for each point at every scale. The three risk areas of tick distribution were used as a backdrop for intersection processing. Ultimately, the ‘cor’ function in R was applied to perform Pearson correlation analysis between the attribute values of the *H. asiaticum* and the fresh feces from Przewalski’s horses at each scale within each risk area.

KDE calculation formula:

4 $${\text{Density}}=\frac{1}{({\text{R}}{)}^{2}}\sum_{{\text{i}}=1}^{{\text{n}}} \left[\frac{3}{\uppi }\cdot {{\text{pop}}}_{{\text{i}}}{\left(1-{\left(\frac{{\text{dist}}_{{\text{i}}}}{\text{R}}\right)}^{2}\right)}^{2}\right]$$

In formula (4), R represents the search radius, ${pop}_{i}$ represents the number of ticks or horses’ fresh feces at point i, and ${dist}_{i}$ represents the distance between the point i and the geographical coordinates.

#### Generalized linear model analysis

Based on the results of multiscale correlation analysis, this study selected the 100 m scale and used the Generalized Linear Model (GLM) to accurately evaluate the impact of Przewalski’s horses and external environmental factors on the *H. asiaticum*. Using the nearest neighbor analysis tool in ArcGIS 10.3, we conducted nearest neighbor analysis on the three water sources and donkey trails. This analysis facilitated the identification of two crucial parameters: the nearest distance to water sources and the nearest distance to donkey trails. Before constructing the GLM, to ensure the model's accuracy and stability, we conducted multicollinearity diagnostics using the Variance Inflation Factor (VIF). The VIF for each explanatory variable was calculated using the 'car' package in R. If VIF ≥ 5, variables were excluded from the model. By constructing a GLM model with the ‘glm’ function in R, we set the attribute values of the *H. asiaticum* at the 100 m scale as the response variable, while considering the attribute values of fresh feces of Przewalski’s horses, the nearest distance to water sources, and the nearest distance to donkey trails as explanatory variables.

## Results

### The ticks sampling sites survey results

During the peak period of ticks, this study encompassed 441sampling sites. Among these, the 219 sampling sites (49.66%) found unfed state adult ticks. A cumulative count of 301 host-seeking ticks was collected, with 275 being *H. asiaticum* (91.36%),identified across all 219 sites. In addition, 14 *Dcrmacentor nuttalli* were collected from 4 sampling sites (4.65%), and 12 *Rhipicephalus microplus* were found across 3 sampling sites (3.99%). The detailed statistics regarding species identification and distribution are presented in Table S1.

### The occurrence rate of *H. asiaticum*

Given that *H. asiaticum* is the predominant species in this region and is present at all tick sampling sites, this study conducted an in-depth analysis of the spatial distribution of the *H. asiaticum*. The occurrence rates of the *H. asiaticum* under different habitats are as follows: donkey trail (65.99%) > water source (55.81%) > grassland (39.04%). Moreover, regions with a high frequency of Przewalski’s horse distribution also exhibit elevated occurrence rates of *H. asiaticum*, characterized by the following sequence: stallion-feces (70.06%) > non-stallion-feces (51.67%) > no-feces (25.97%). In the following sites, the occurrence rate of *H. asiaticum* exceeded 50%: stallion feces at water source (77.27%) > stallion feces at donkey trail (72.88%) > non-stallion feces at grassland (53.41%) > stallion feces at grassland (51.85%) > non-stallion feces at donkey trail (50.00%) (Table 1).

**Table 1:** Table of *H. asiaticum* collection in Kalamaili Nature Reserve (KNR)

Indicators	Donkey Trail		Grassland		Water Source		In total	
	N	P	N	P	N	P	N	P
Stallion-Feces	118	72.88	27	51.85	22	77.27	167	70.06
Non-stallion-Feces	18	50.00	88	53.41	14	42.86	120	51.67
No-Feces	11	18.18	136	27.21	7	14.29	154	25.97
In total	147	65.99	251	39.04	43	55.81	441	49.66

*N* The number of sampling site, *P* The occurrence rate of ticks (%). Donkey trail: Refers to the habitual pathways or trails traversed by local equids; Grassland: Denotes the foraging regions frequented by Przewalski's horses; Water source: Refers to the natural water sources where Przewalski's horses commonly quench their thirst. (Fig. 1)

### *H. asiaticum* occurrence rate and Przewalski’s horse dung parameters

**Fig. 2:**
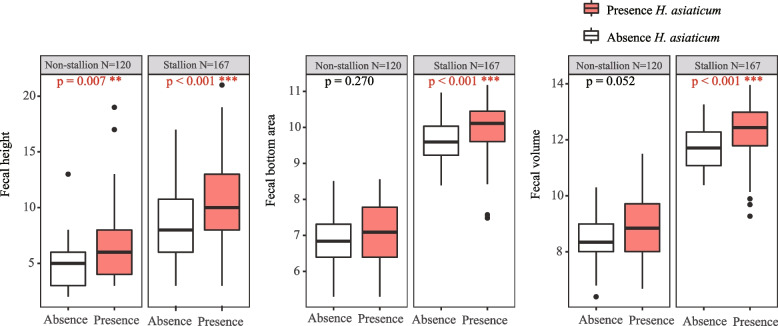
The T-test analysis of Przewalski's horses fecal size differences in presence and absence of *H. asiaticum*

Pearson correlation analysis demonstrated a positive correlation between the size of Przewalski’s horse feces and the number of the *H. asiaticum*. The correlation coefficients are as follows: fecal height (*r*_*(287)*_ = 0.401, *P* < 0.001), fecal bottom area (*r*_*(287)*_ = 0.328, *P* < 0.001), fecal volume (*r*_*(287)*_ = 0.369, *P* < 0.001). The T-test analysis revealed that the *H. asiaticum* prefer to conceal themselves near larger piles of Przewalski’s horse feces (Fig. 2). Specifically, stallion feces showed significant differences in the height (t_(167)_ = 3.659, *P* < 0.001), bottom area (t_(167)_ = 2.752, *P* < 0.001), and volume (t_(167)_ = 3.846, *P* < 0.001), while non-stallion-feces only showed significant difference in height (t_(120)_ = 3.000, *P* < 0.01).

**Fig. 3:**
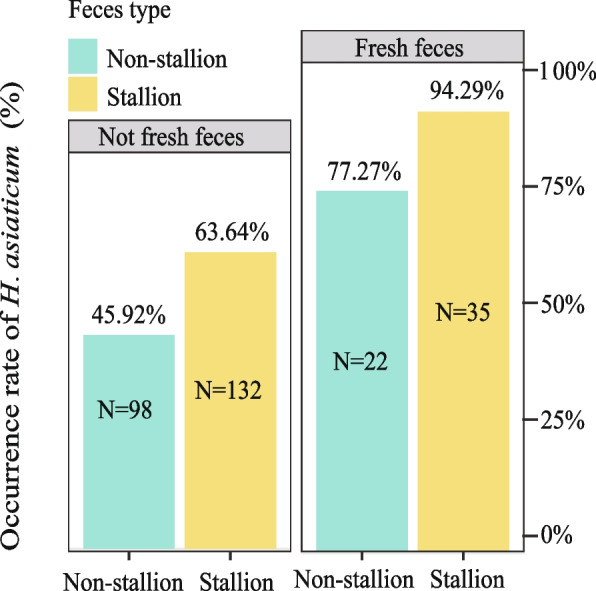
The correlation between humidity of Przewalski's horse feces and the occurrence rate of *H. asiaticum*

The study found that the fresher the Przewalski’s horse feces, the higher the occurrence rate of the *H. asiaticum*, and the occurrence rates of ticks near stallion feces is generally higher than near non-stallion feces (Fig. 3). The occurrence rates of the *H. asiaticum* was distributed as follows: high humidity of stallion feces (94.29%) > high humidity of non-stallion feces (77.27%) > low humidity of stallion feces (63.64%) > low humidity of non-stallion feces (45.92%).

### Three risk area of *H. asiaticum*

**Fig. 4:**
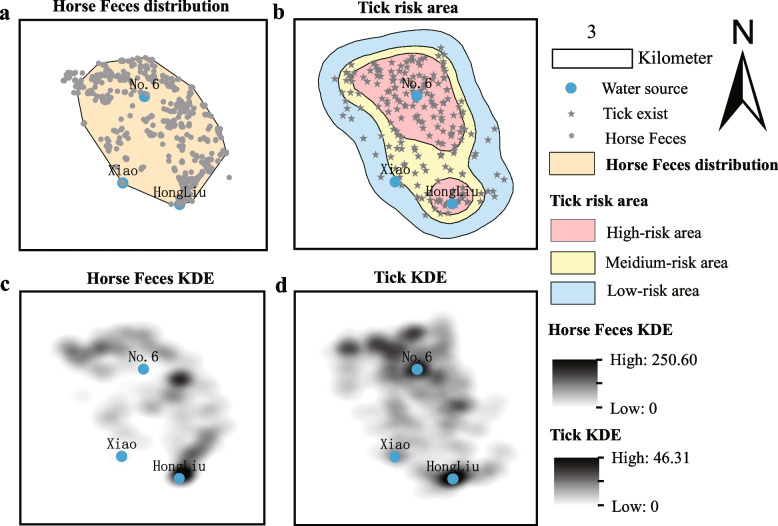
Distribution of Przewalski’s horse fresh feces and *H. asiaticum*. **a** Distribution area of Przewalski’s horse fresh feces. **b** Three risk areas of *H. asiaticum*. **c** Kernel Density Estimation (KDE) distribution of fresh feces from Przewalski’s horses. **d** KDE distribution of *H. asiaticum*

This analysis used the data from 219 sampling sites of ticks and the 669 locations of Przewalski's horse fresh feces. (Fig. 4a, b). The area covered by fresh feces from Przewalski's horses in the survey region was determined to be 14.31 km^2^, as calculated by the 95% MCP method (Fig. 4a). We categorized the risk areas for *H. asiaticum* into high, medium, and low categories based on 50% FKE, 75% FKE, and 95% FKE thresholds. Subsequent analysis revealed a positive correlation between the risk levels of ticks and the overlap rate with the distribution of Przewalski's horse (Table 2). Specifically, the high-risk area for *H. asiaticum* was 8.00 km^2^, of which 90.25% overlapped with the distribution of Przewalski's horses, covering an area of 7.22 km^2^ (Fig. 4b, Table 2); the medium-risk area was 7.04 km^2^, with 33.79% overlap rate, overlap area covering 4.80 km^2^ (Fig. 4b, Table 2); and the low-risk area was 9.96 km^2^, with 23.09% overlap rate, overlap area covering 2.30 km^2^ (Fig. 4b, Table 2).

**Table 2:** Statistical table of the overlap between the three risk areas of *H. asiaticum* and the distribution of Przewalski's horse fresh feces

Risk level classification	Area	Overlap area	Overlap ratio
High-risk area 50%FKE	8.00	7.22	90.25
Medium-risk area 75%FKE	7.04	4.80	33.79
Low-risk area 95%FKE	9.96	2.30	23.09

*FKE* Fixed Kernel Estimation; Overlap area (km^2^), Overlap area between the distribution of fresh feces from Przewalski's horses and the risk areas of *H. asiaticum*. Overlap ratio (%), Probability of overlap between the distribution of fresh feces from Przewalski's horses and the risk areas of *H. asiaticum*

### Spatial autocorrelation index Moran's *I* of *H. asiaticum* and Przewalski's horse fresh feces

In the three designated risk areas, we observed that the distribution of *H. asiaticum* and horse fresh feces exhibited an extremely high degree of spatial autocorrelation, with Moran's *I* values approaching 1 (Table 3). The Z-score values further substantiated the statistical significance of this aggregation pattern, thereby greatly diminishing the probability of a random distribution (*P* < 0.001). In the high-risk area, Moran's *I* value was 0.998 (Z = 16.499, *P* < 0.001) and similarly, in the medium-risk area, Moran's *I* was also 0.998 (Z = 8.968, *P* < 0.001). This indicates a very strong spatial clustering of *H. asiaticum* and horse fresh feces in both high and medium-risk areas. In the low-risk area, the Moran's *I* value was slightly lower at 0.994 (Z = 8.335, *P* < 0.001), still indicating a significant spatial aggregation trend of *H. asiaticum* and horse fresh feces (Table 3).

**Table 3:** Statistical table of the spatial autocorrelation index Moran's *I* for *H. asiaticum* and Przewalski's horse fresh feces

	Moran’s *I*	Z score	Expectation	Variance	*p* value	Clustered
High	0.998	16.449	-0.002	0.004	< 0.001***	Yes
Medium	0.998	8.968	-0.008	0.013	< 0.001***	Yes
Low	0.994	8.335	-0.009	0.015	< 0.001***	Yes

*** indicates significance at the 0.001 level

### Kernel density estimation and multiscale correlation analysis of *H. asiaticum* and Przewalski's horse fresh feces

The KDE analysis indicated that the distribution of fresh feces from Przewalski's horses is predominantly concentrated to the north of major water sources, including HongLiu and No. 6 (Fig. 4c). Similarly, *H. asiaticum* were also predominantly found in the areas near these water sources (Fig. 4d). To quantify the correlation between *H. asiaticum* and horse fresh feces at different scales, this study conducted Pearson correlation analysis at distances at distances of 100 m, 250 m, 500 m, 750 m, and 1000 m (Table 4). The specific distribution sites at these five scales are presented in Fig. S5. At the 100 m scale, a positive correlated was observed across all risk areas, with the strength of the correlation diminishing in the order of high, medium, and low. This suggests that the presence of horse fresh feces may be more closely correlation with the distribution of *H. asiaticum* at smaller spatial scales. However, at larger scales such as 500 m, 750 m, and 1000 m, the variability in correlations indicates that factors other than horse fresh feces could be influencing the distribution of *H. asiaticum*, particularly noted in the negative correlations observed in medium-risk areas at 500 m and 1000 m scales (Table 4).

**Table 4:** Statistical table of Pearson correlation coefficients of *H. asiaticum* and Przewalski's horse fresh feces under five scales

	100 m	250 m	500 m	750 m	1000 m
High	0.323	0.283	0.193	0.529	0.188
Medium	0.238	0.293	-0.024	0.662	-0.514
Low	0.145	0.130	0.155	0.062	0.202

100 m、250 m、500 m、750 m、1000 m represents different scales

### Comprehensive analysis using generalized linear model

In this study, we used a GLM to unveil the significant correlations between the distribution of *H. asiaticum* and three pivotal factors: the distribution of the horse fresh feces; nearest distance to water sources; nearest distance to donkey trails (Table 5). The multicollinearity analysis results show that the VIF for the horse fresh feces is 1.134, for the nearest distance to water sources is 1.048, and for the nearest distance to donkey trails is 1.143. The VIF values of these three variables are close to 1, indicating almost no linear correlation between them, making them suitable as explanatory variables in the GLM. The intercept of the GLM is 13.536 (T = 44.170, *P* < 0.001). Notably, the distribution of fresh feces from Przewalski’s horses showed a significant positive correlation with the distribution of *H. asiaticum* (T = 27.980, *P* < 0.001), suggesting that accumulations of horse feces can attract the ticks. Conversely, the proximity to water sources exhibited a negative correlation with the tick distribution (T = -14.940, *P* < 0.001), similar to the proximity to donkey trails (T = -25.630, *P* < 0.001), indicating that closer proximity to water sources and donkey trails increases the likelihood of encountering *H. asiaticum* (Table 5).

**Table 5:** Statistical table of comprehensive generalized linear model analysis

Coefficients	Estimate	Std. error	T value	*p* value
Intercept	13.536	0.306	44.170	< 0.001***
Horse fresh feces	0.0984	0.004	27.980	< 0.001***
Nearest distance to water sources	-0.008	0.001	-14.940	< 0.001***
Nearest distance to donkey trails	-0.005	0.001	-25.630	< 0.001***

*** indicates significance at the 0.001 level

## Discussion

This study aims to delve into the impact of the reintroduction of Przewalski’s horses on the parasites in the desert grassland ecosystem of the KNR, with a focus on analyzing the influence of their distribution patterns on *H. asiaticum*. The reintroduction of Przewalski’s horses, an endangered species, not only represents a crucial measure for biodiversity conservation but also significantly affects the structure of the local parasitic community, which has been confirmed by previous studies [10–12]. Although the primary intent of reintroduction is to protect or restore ecosystems, it may result in unintended consequences [38, 39, 51]. This study is the first to identify a positive correlation between the distribution of Przewalski’s horse feces and the spatial distribution patterns of the *H. asiaticum*, providing new insights into the mechanisms by which the reintroduction of Przewalski’s horses impacts the parasites in desert grassland ecosystems. This finding is consistent with studies from other regions regarding the relationship between reintroduced species and ticks. For example, in Kentucky, reintroduced elk have turned into hosts for various ticks, potentially expanding their distribution [36]. Similarly, in Japan, an increase in the population of wildlife has coincided with an increase of ticks [37]. Therefore, this study points out that even introducing animals for conservation purposes could inadvertently facilitate the spread of ticks and their pathogen carriers [36, 37].

The occurrence rates of the *H. asiaticum* and its relationship with Przewalski’s horse feces constitute the central focus of this study. Previous research has revealed that host feces significantly impact the spatial distribution of parasites [12, 52, 53], evidenced by the parasites’ inclination to gather in the areas proximate to the host’s feces [11, 12, 15]. In light of this context, and considering the tendency of Przewalski’s horses in the KNR to congregate in family units [7, 12, 54], as well as the proactive host-seeking behavior of *H. asiaticum* [15], this study posits that zones where Przewalski’s horses frequently defecate are more likely to encounter ticks in an unfed state. This hypothesis was supported by field data indicating that the *H. asiaticum* was found significantly more frequently in proximity to the Przewalski’s horse feces than in areas without feces. Subsequent analysis elucidated a positive correlation between the size and freshness of the horse feces and the occurrence rates of the *H. asiaticum*. This phenomenon suggests that Przewalski’s horse feces may provide an important survival environment for the *H. asiaticum* [55, 56]. Significantly, this spatial association is not limited to the *H. asiaticum*, other studies have also shown that zones of frequent defecation by Przewalski’s horses are considered high-risk for obligate parasites such as horse stomach flies [10–12]. These findings highlight the necessity of integrating a comprehensive consideration of the multifaceted impacts of reintroducing endangered species on other species within local ecosystems when formulating biodiversity conservation strategies and ecosystem management measures.

In ecological research, animal feces are considered a valuable ecological indicator for discerning their behavioral patterns and spatial distribution [47, 48]. Previous studies have confirmed a substantial correlation between the distribution of ungulates and ticks, as exemplified by the findings of Qviller et al. [47], who discovered a tight spatial association between the distribution of the red deer and the *Ixodes ricinis* in Norway. Similarly, Schulze et al. [57] observed a close relationship between the distribution of the white-tailed deer and the *Amblyomus* ticks in America. However, due to the numerous challenges of field surveys and the limitations in data collection, this study prefers to use fresh feces as an indirect indicator of Przewalski's horses’ activity range. The *H. asiaticum*, an ectoparasite that actively seeks hosts based on host scent [15, 28, 30], examining the distribution of Przewalski's horse fecal feces can better illustrate the spatial correlation between the host animals and ticks. Furthermore, initial investigations in this study found a relatively high prevalence of ticks near fresh feces of Przewalski's horses, confirming that data on fresh feces from synchronized surveys can serve as an indirect indicator of the spatial association between Przewalski's horses and the *H. asiaticum.*

Przewalski's horses have a social structure based on family groups, typically consisting of one stallion, three to four mares, and their foals [9, 58]. Different genders of Przewalski's horses may display unique activity patterns and territorial marking behaviors, leading to their feces containing different chemical information [32, 43, 59], which could affect their attractiveness to ticks and influence tick distribution. In this study, fecal samples were differentiated by observing that stallions and non-stallions defecate in different locations. Specifically, mares and foals tend to defecate randomly on grasslands, whereas stallions prefer to repeatedly defecate in the central areas of their family group’s territory [54, 58]. The results showed a higher probability of ticks near stallion feces than feces of the non-stallions, with the larger feces showing a higher occurrence rate of ticks. This suggests that the spatial distribution of ticks in this region may be related to the volume of Przewalski's horse feces and their gender-specific behaviors.

This finding provides a basis for future research on how gender difference in Przewalski's horses could influence tick distribution. Moreover, the widespread *E. hemionus* and *G. subgutturosa* in the reserve exhibit a more dispersed distribution due to their lower dependency on water and higher alertness, leading to the observation of typically drier feces [60, 61], which are less suitable for tick spatial distribution studies. According to Zhang et al. [12] and Huang et al. [11], fresh feces of these species are rare in our study area, thus their direct impact on the study results is limited. This supports the study's focus on Przewalski's horses, an endangered reintroduced species, and largely excludes the impact of other local wildlife in the study area on the spatial distribution of ticks.

Before the reintroduction of the Przewalski’s horses, the KNR had not shown a high correlation between the distribution of ungulates and ticks [16]. Research suggests that post-reintroduction, the increased density of host feces from Przewalski's horses may have amplified the spread and reproduction of parasite populations, thereby accelerated the transmission patterns of parasites and potentially altered the ecological chain relationships between local hosts and parasites [12]. Therefore, this research focuses on analyzing the distribution relationship between the Przewalski's horses and the *H. asiaticum*, and it quantifies the risk levels of *H. asiaticum* in the surveyed area. The MCP algorithm was deployed to estimate the distribution area of Przewalski's horses. Although a 100% MCP can include all GPS sites, it may overestimate the area due to extreme points [62]. Consequently, a 95% MCP was chosen to exclude the impact of extreme points. This method is in line with methods previously employed by researchers to determine the home range of Przewalski's horses [7, 62]. The FKE method enhances the MCP by smoothing the distribution area calculation through correcting extreme points [12, 63] For this study, 50% FKE, 75% FKE, and 95% FKE were utilized as thresholds to delineate high, medium, and low-risk areas, respectively, in order to quantify the risk areas of the *H. asiaticum*. Through analysis of the overlap rates, it was observed that areas with the fresh feces form the Przewalski's horses overlap with high-risk areas for the *H. asiaticum* by more than 90%. This suggests that Przewalski's horses are consistently exposed to tick-infested environments within their activity areas. Moreover, high-risk areas frequently correspond with areas where Przewalski's horses are commonly active and defecate, which aligns with our conclusions drawn from spatial autocorrelation analysis. These methods of analysis approaches allow us to reveal the spatial relationship between the distribution of the *H. asiaticum* and horse fresh feces, indicating that the tick distribution is not random but closely related to the spatial distribution of horse fresh feces.

The water sources and grasslands are crucial factors for the survival of ungulate species in arid and desert regions [31, 40]. The strong reliance of Przewalski’s horses on these water sources often directs their frequent use of donkey trails near these areas, consequently resulting in more severe occurrences of the *H. asiaticum* in such areas [11, 12, 31]. Grassland areas, being vital habitats for wild ungulates, may exhibit a comparatively lower presence of ticks. This phenomenon could be due to Przewalski’s horses lingering and being active over extended periods in these areas [32], leading to a higher number of the *H. asiaticum* successfully attaching to the horses. Therefore, by focusing the analysis on three factors associated with a higher occurrence rate of ticks — the presence of fresh Przewalski's horse feces, proximity to water sources, and closeness to trails used by donkeys — we can more effectively assess the impact of hosts and ticks distribution. To address potential multicollinearity among these variables, VIF values were calculated and found to be below 5, signifying an absence of significant multicollinearity. This indicates that these factors can serve as independent explanatory variables in the model. This study employed a combination of KDE and GLM to comprehensively analyze the spatial association between fresh feces of Przewalski’s horses and the *H. asiaticum* across various habitats. The KDE method revealed a significant spatial correlation between the *H. asiaticum* and fresh feces of Przewalski’s horses within a 100 m scale, conforming to the established criteria for categorizing areas into high, medium, and low risk levels [47, 63–65]. The GLM analysis further indicated that the distribution of the *H. asiaticum* is positively correlated with the presence of fresh feces from Przewalski’s horses. Moreover, it revealed a negative correlation with the proximity to water sources and donkey trails. This implies that the higher the abundance of fresh feces from Przewalski’s horses, and the closer the proximity to water sources and donkey trails, the higher the probability of the distribution of the *H. asiaticum*. This not only intensifies the threat of the *H. asiaticum* in these regions but also exposes other wildlife species, including the Mongolian wild ass and the goose-throated gazelle, which visit these water sources to drink, to increased risks of parasitic infestations [31, 33]. Due to the unique digestive system of Przewalski’s horse, it is highly dependent on water sources during the spring and summer tick peak period [10, 16, 33]. Long-term gathering near water sources may lead to the risk of infestation with the *H. asiaticum* in these areas, which will affect the success rate of its reintroduction process and aggravate the expansion of tick-borne diseases in the desert steppe ecosystem [12, 31, 33].

The reintroduction of wild animals often results in them feeling unfamiliar with their post-release environment, leading to more limited spatial movement [8, 9, 51, 66]. As the reintroduction process in the KNR region advances, the increasing activity range of Przewalski’s horses may result in the spread of parasites to wider areas, posing a potential threat to the health of other local species [7, 10, 12]. Furthermore, due to the reintroduced species may lack sufficient resistance to endemic parasites, which could lead to more severe infestations compared to other species [51]. This study using the reintroduction of Przewalski’s horses in the KNR as a case study, provides insights for scholars and managers. It emphasizes that when Przewalski’s horses establish a stable presence in an area, it invariably affects the local parasitic situation [11, 12]. While Przewalski's horses fall victim to parasitic infections, their presence also transforms the area into the high-risk areas for parasites [12]. The purpose of reintroduction is to protect endangered species or associated species to maintain their population levels. However, concentrating solely on population growth without considering the stability of the entire ecosystem can disturb the local ecology. In extreme cases, this may impact the health and survival activities of wildlife in the entire region [51, 67–69]. Therefore, through this study, we suggest that relevant departments, when formulating reintroduction policies, should comprehensively consider and assess the impact of new animal introductions on ecosystems. They should implement measures to mitigate these potential problems, such as setting tick traps in the animals’ active areas to monitor and manage parasite transmission and creating additional water sources to decrease the animals’ density. These measures could indirectly control parasite spread, improve living conditions for the Przewalski's horses, and reduce potential risk of spillover and spillback of zoonotic diseases.

## Conclusions

With the reintroduction of the endangered Przewalski's horses to the KNR, there has been a sustained increase in both its population size and the frequency of their activities within its habitat. This developing trend of species reintroduction, aimed at maintaining biodiversity, may potentially disturb the established ecological balance between hosts and parasites in local ecosystems. Additionally, it could inadvertently lead to the introduction of other non-native species or disease vectors, further complicating ecological interactions. This study has found that the spatial utilization characteristics of Przewalski's horses in this region would have a significant impact on the distribution of the *H. asiaticum*. The horses' marked reliance on particular habitats, like water sources and donkey trails, leads to a heightened density of the *H. asiaticum*. This not only escalates the risk of parasitic infestation risk for the horses themselves but also presents a potential threat to other wildlife reliant on the same water sources. Additionally, the potential absence of immunity to local parasites in the reintroduced horses could intensify parasitic concerns within the reintroduction areas. Consequently, systematic planning in wildlife conservation and consideration of the complex effects of reintroduced animals on existing ecosystems are vital in maintaining ecological balance and protecting biodiversity. Through comprehensive management and prevention strategies, reintroduction can be ensured as a beneficial approach for biodiversity conservation and ecological restoration, rather than becoming a new threat to ecosystem stability.

### Supplementary Information

Additional file 1: Fig. S1 The pictures of the *H. asiaticum* bites on the abdomen of Przewalski's horses in Kalamaili Nature Reserve (KNR).pdf

Additional file 2: Fig. S2 Accidental discovery of naturally detached engorged female *H. asiaticum* near stallion feces on donkey trails.pdf

Additional file 3: Fig. S3 The Method of tick sampling sites under three habitat types.pdf

Additional file 4: Fig. S4 Three types of activity traces of Przewalski's horses: stallion feces, non-stallion feces, no feces.pdf

Additional file 5: Fig. S5 Distribution of grids and points at five different scales.pdf

Additional file 6: Table S1 The statistics of ticks’ identification and distribution at sampling sites.xlsx

## Data Availability

All data generated or analyzed during this study are included in this published article and its additional information files.

## Abbreviations

KNR: Kalamaili Nature Reserve
MCP: Minimum Convex Polygon
FKE: Fixed Kernel Estimation
KDE: Kernel Density Estimation
VIF: Variance Inflation Factor
