# Supplementary material for: Biological response to Przewalski’s horse reintroduction in native desert grasslands: a case study on the spatial analysis of ticks
Source: BMC Ecol Evol. 2024 May 11;24:61. doi: 10.1186/s12862-024-02252-z (PMC11088120; doi:10.1186/s12862-024-02252-z)

Additional file 1: FIG. S1.

The pictures of the *H. asiaticum* bites on the abdomen of Przewalski's horses in KNR

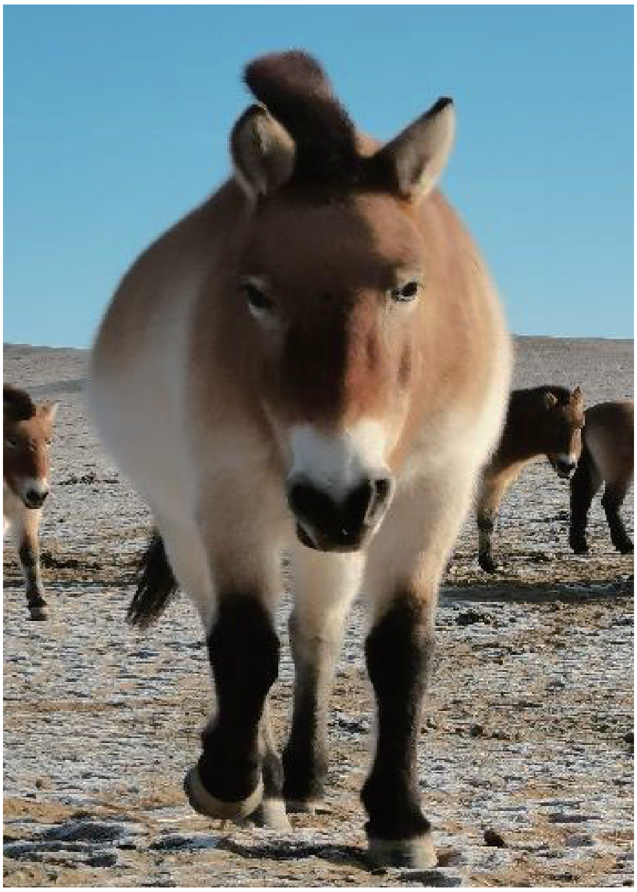

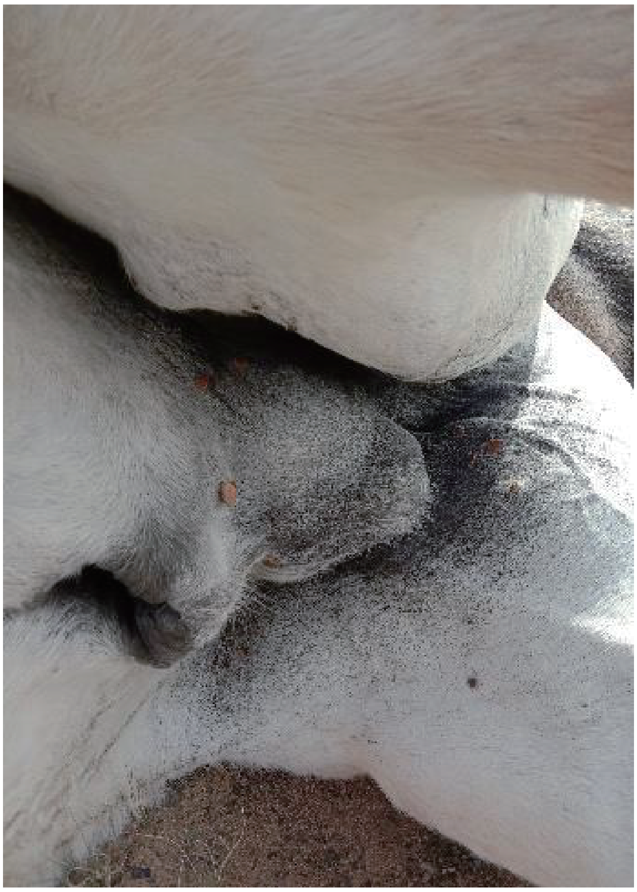

Supplement: Supplementary file 1 — Additional file 1: Fig. S1 The pictures of the H. asiaticum bites on the abdomen of Przewalski's horses in Kalamaili Nature Reserve (KNR).pdf [file 12862_2024_2252_MOESM1_ESM.pdf]
