# Supplementary material for: Biological response to Przewalski’s horse reintroduction in native desert grasslands: a case study on the spatial analysis of ticks
Source: BMC Ecol Evol. 2024 May 11;24:61. doi: 10.1186/s12862-024-02252-z (PMC11088120; doi:10.1186/s12862-024-02252-z)

Additional file 2: FIG. S2

Donkey trail

Accidental discovery of naturally detached engorged female *H. asiaticum* near stallion feces on donkey trail

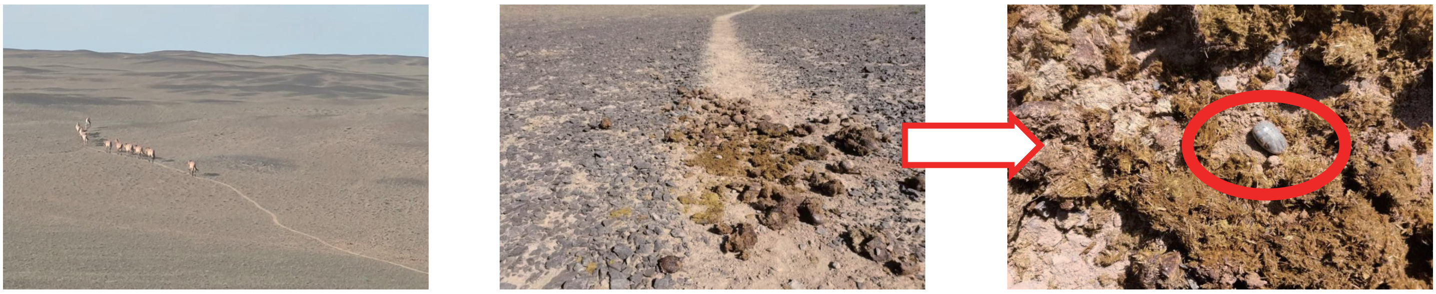

Supplement: Supplementary file 2 — Additional file 2: Fig. S2 Accidental discovery of naturally detached engorged female H. asiaticum near stallion feces on donkey trails.pdf [file 12862_2024_2252_MOESM2_ESM.pdf]
