# Supplementary material for: Biological response to Przewalski’s horse reintroduction in native desert grasslands: a case study on the spatial analysis of ticks
Source: BMC Ecol Evol. 2024 May 11;24:61. doi: 10.1186/s12862-024-02252-z (PMC11088120; doi:10.1186/s12862-024-02252-z)
Supplement: Supplementary file 3 — Additional file 3: Fig. S3 The Method of tick sampling sites under three habitat types.pdf [file 12862_2024_2252_MOESM3_ESM.pdf]

滚滚长江东逝水

Additional file 3: FIG. S3

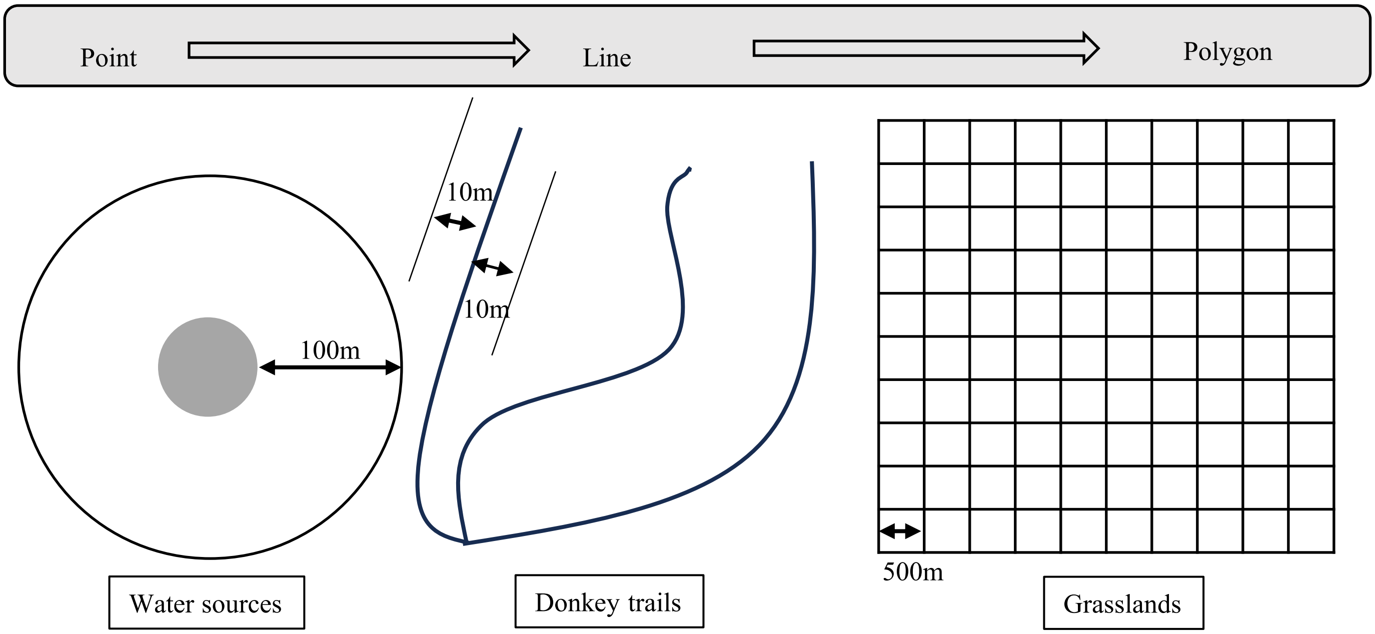

This study, focused on the activity characteristics of Przewalski's horses, established tick sampling sites in three distinct habitats: water sources, donkey trails, and grasslands. Within these habitats, sampling sites were differentiated based on the presence or absence of feces, including stallion feces, non-stallion feces and no feces, to ensure a comprehensive collection of tick data. The specifics include:

Water Sources: A buffer zone of 100 meters was established around each water source. Within this zone, 20 tick sampling sites were randomly selected, totaling 60 tick sampling sites .

Donkey trails: With each water source as the center, three donkey trails were selected, and a total of 81 tick sampling sites were surveyed along these trails.

Grasslands: The grassland area was divided into 100 grids, each measuring 500m × 500m, and tick surveys were conducted within these grids, totaling 300 tick sampling sites.

However, due to the geographical overlap of donkey trails with grasslands and water sources, to align with the actual survey results and ensure the accuracy of data interpretation, this study reclassified sample points in the water source and grassland areas that were closer to donkey trails into the donkey trails category.
