# Supplementary figures and images for: Biological response to Przewalski’s horse reintroduction in native desert grasslands: a case study on the spatial analysis of ticks
Source: BMC Ecol Evol. 2024 May 11;24:61. doi: 10.1186/s12862-024-02252-z (PMC11088120; doi:10.1186/s12862-024-02252-z)

Additional file: FIG. S4

Stallion feces

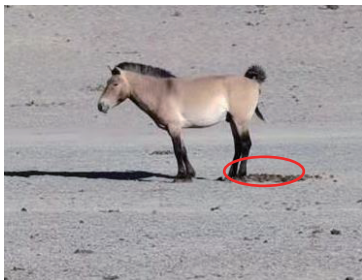

Non-stallion feces

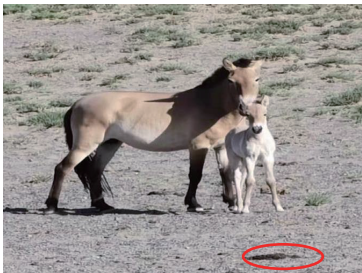

No feces

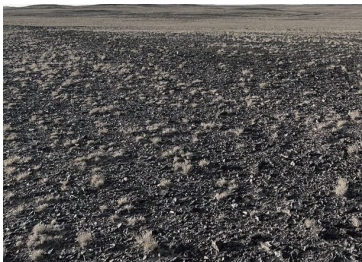

Supplement: Supplementary file 4 — Additional file 4: Fig. S4 Three types of activity traces of Przewalski's horses: stallion feces, non-stallion feces, no feces.pdf [file 12862_2024_2252_MOESM4_ESM.pdf]

Additional file 5: FIG. S5.

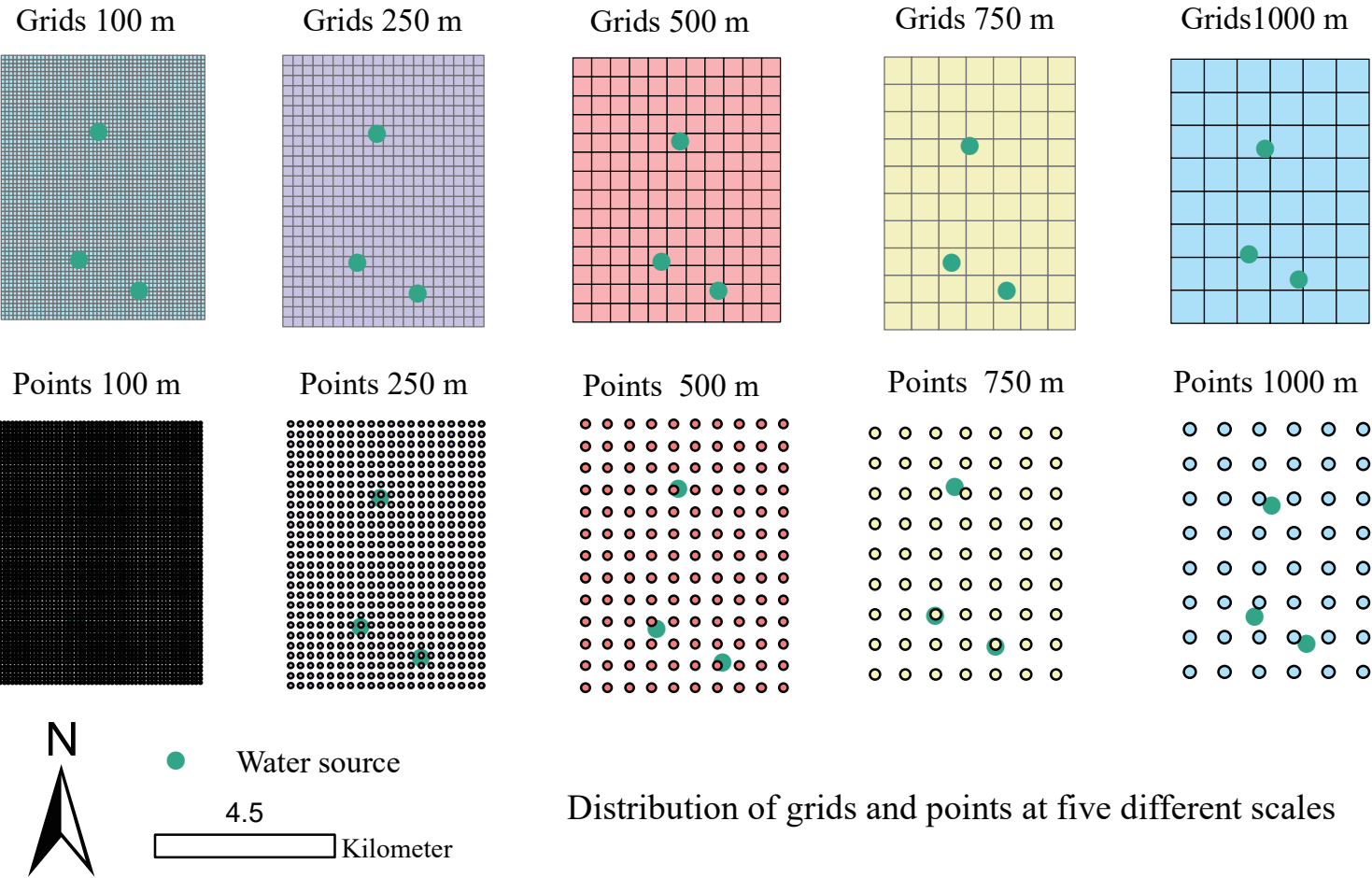

Supplement: Supplementary file 5 — Additional file 5: Fig. S5 Distribution of grids and points at five different scales.pdf [file 12862_2024_2252_MOESM5_ESM.pdf]
